# Strategies for STEM and SEMG applications in clinical practice-lessons from the past

**DOI:** 10.3389/fresc.2024.1500316

**Published:** 2024-12-05

**Authors:** Sanjeev Gupta, Saanvi Aggarwal

**Affiliations:** ^1^ Department of Physiotherapy, Gurugram University, Gurugram, India; ^2^ Sri Padmavathi Medical College for Women, Tirupati, India

**Keywords:** sEMG (surface electromyogram), STEM—science technology engineering mathematics, physiotherapy, clinical practice, policy making in education

*“In all developmental journeys, change in behaviour is enforced first, before one appreciates it as a useful habit”*

## 1 Introduction

Acceptance of sEMG is an issue of paradigm shift. It puts clinician in an uncomfortable position to choose between tradition-innovation, convenience-diligence, quick-correct, etc. There is behavioural inertia that is slowing the acceptance of sEMG. Paradigm shifts of recent times are “recycling of plastic”, “shifting from combustion engines”, “shift from thermal power to hydro/solar”, “standardization of Health establishments”, “annual to semester calendar”, “digitization of records/processes” etc. All these changes were advantageous, but they didn't happen easily. An orchestrated policy, backed by qualified “techno-bureaucratic will” realized such paradigm shifts that are now norms. Generally, clinicians are technology laggards and issues of resources availability are not directly under clinicians’ control. Administrators of healthcare are the controllers of “resource allocation” (systems, space, finance, manpower and support services). They are executives articulating between management's interests (primarily) and demands of clinicians. They are sensitive to profit enhancing propositions and statutory compliances. Surface EMG fails on both of these attributes. It is not mandated by norms and its cost effectiveness studies are limited (1). Adopting sEMG implies a significant learning effort (undesirable to clinician) and increased resources (undesirable to administration). Hence well-known limitations of sEMG, reported in research studies, are stressed to avoid dealing with the subject. This is a common approach to distract attention from root-cause. Actual issues are rarely reported objectively. A decisive policy is needed to upgrade teaching and outdated clinical practice, as discussed in literature (2–4).

The following sections list and describe a number of proposed actions and the role of the stakeholders.

## 2 Proposed actions

The first author has been directly involved in Indian Government policy-making for implementing reforms in academic syllabi, accreditation, internship-training, assessing public/private institutions, career enhancement schemes, regulatory compliance, etc. and has observed great differences between what health managers say in research/reports and how they behave in practice. Vital initiatives required to establish sEMG in the order of importance are:

### 2.1 Policy making

sEMG based quantitative outcome (e.g., RMS) measures are not exploited so far in an appropriate manner. Orchestrated efforts is required by researchers & industry to press Statutory Councils; NCAHP (5), WCPT (6), APTA (7), NHS (8) HCPC (9) (Appendix 1) to exercise their scope & advocacy to promote applications of sEMG as a clinical standard. EMG-RMS, onset and muscle-activation times, muscle fibre conduction velocity, spectral parameters, neural-drive, EEG-EMG coherence, etc. These quantities should be stipulated by agencies as essential outcome measures for model clinical assessment as proposed by the ISEK-JEK tutorials, best practices, and consensus papers (6–14). Agencies should prescribe sEMG as a full semester subject associated to Biomechanics and Electrophysiology in PT programs. Specialized diploma should also be introduced for practitioners. Once sEMG is a requirement to comply with, clinicians will initiate efforts to learn/apply it and they will find answers to many challenges. Professional organizations should offer awards/scholarships in sEMG and promote sEMG workshops as commendably done by ISEK (10) through the publication of a series of Tutorials (11–14) and Consensus paper (2, 15, 16).

### 2.2 Role of industry

Besides rampant marketing, teaching maybe expected by sEMG device companies. For most of the new technologies there is no market yet. Commercial companies need to invest for long-term, cut costs and support research.

### 2.3 Integration

Of established PT training with sEMG should be brainstormed for managing production/ancillary costs and mustering survival of business. The phenomenal success of smart phone merging many devices into one is a relevant example here. Developing simpler devices with preset modes and default settings is suggested. Employing physiotherapists in sEMG device companies will make students interested in sEMG learning during their academic training.

## 3 Strategic role of physiotherapists

In India, and many other countries, PTs undergo 4 years of UG education (BS) followed by 2 years of PG education (MS) and a PhD. They do clinical diagnosis (5) and measurement of muscles/movement and should be educated to record, process and interpret sEMG signals as expressions of neuromuscular control. Physiotherapists are at the pivot of creating knowledge about movements, on one side, and applying STEM methodologies for patient-care, on the other side. PTs are vital in assessing and monitoring rehabilitation of all movement disorders. With the phenomenal improvement in research and academics, PTs are the most efficient lever to optimize therapies and measure outcomes in a rehab team. The majority of physicians/surgeons endorse the role of PTs as vital in early and effective functional restoration of movement. PTs are widely accepted in society and patient community at large. Recognized as pain relieving and activity restoration specialist by non-drug methods PTs are welcome saviours especially for managing chronic disorders (5). PTs are among the few health operators who exploit electronic instrumentation such as electrical stimulation, ultrasonic, diathermy and are well versed with use and safe handling of electrical equipment. Clinical monitoring of anomalies by evoked responses like Faradic-Galvanic testing, chronaxie, rheobase, strength-duration curve etc. are age-old in PT. Additional training of physiotherapists may include sEMG signal processing, electrode placement, data acquisition, feature extraction and interpretation, sources of errors, etc.

The precise role of PTs in this regard can be categorized in the following strata that also apply to any other technological field:

### 3.1 Infrastructure requirement

It should be made mandatory to have sEMG instrumentation for PT labs, with mandatory experiments in the academic curriculum (1, 17, 18).

### 3.2 Curriculum

The current (5) curriculum in physiotherapy is not suitable to produce a clinical figure with proper competence in sEMG and other assessment techniques. There should be a dedicated subject in UG and a specialty in PG studies (including STEM topics), with proportionate weight in examination/evaluation. Teaching sEMG practice at the UG level is a practical approach to avoid “inertia to embrace technology” that is later observed in experienced PTs. There is a need to introduce a minimum 10–12 credits course on sEMG at UG level and PG degree in clinical sEMG. A syllabus for such a course is proposed in another paper of this collection.

### 3.3 Clinical practice

A policy is required on Standard operating (clinical) Procedures to make sEMG reporting mandatory to document clinical assessment and monitoring.

### 3.4 Research thrust

sEMG research in PG and PhD programs should be promoted by offering grants, seed money, journal subscriptions, etc.

### 3.5 Faculty development programs

A policy should be set on mandatory credit requirement to attend sEMG-related workshops.

### 3.6 Career enhancements

Avenues in the form of “industry-reward” for exemplary sEMG practice.

## 4 Target group

The largest target for sEMG knowledge dissemination are UG students. Their approach is positive and, if motivated, they do show keenness towards sEMG. Students show interest in modern instrumentation and their capacity to grasp STEM technology is higher than physiotherapists’ who focus on patient loads. The tendency to resist changes in clinical practices is less likely in students, especially if it is linked with their academic-performance/curricular-credits. sEMG instrumentation can be made available in institutions whereby more students can learn at one center. Clinicians show less interest in CME on sEMG as they never learned its scope/relevance in practice.

## 5 Translation strategy

Three pronged “EMG strategy” should be applied to present sEMG knowledge and instrumentation to the learner's mind: (1) Educate, (2) Mandate, and (3) Grow.

### 5.1 Educate

A combination of appropriate STEM concepts with practical sessions at para-clinical UG education, is necessary to lay foundation in STEM and sEMG practice. This is the stage where the matrix of clinical practice is being laid in a learner mindset. Clinical practice is a permanent fabric whose individual threads are woven in time at the UG and PG learning. This fabric remains permanent throughout the future practice. What they learn in their later clinical life introduces additional knowledge that is embroidered on this permanent fabric. Based upon this analogy, the most appropriate level to introduce sEMG is the UG education (para-clinical learning stage). Content of STEM and sEMG should be smartly planned to avoid aversion to it by the learner. STEM may be introduced in nutshell with relevant clinical examples. STEM concepts may be co-laterally taught during “hands-on filtering/feature-extraction sessions”, when the learner is more likely to understand them due to higher motivation derived by the need of applying them.

### 5.2 Mandate

Knowledge and application of sEMG should be made mandatory for clinical practice by statutory measures. There should be evaluation of STEM and sEMG knowledge, as well as emphasis on quantitative reporting of muscle-movement performance, mandatory sEMG clinical practice, student exchange programs with the centers of excellence in field of sEMG and mandatory attendance to sEMG seminars/conferences. There must be mention of sEMG application at all possible stages of protocols for assessing treatment. There is a need to anchor sEMG practice in clinician's regular work.

### 5.3 Grow

Collateral methods of learning is an unexploited area in sEMG education and promotion. A new strategy of learning can be based on the fact that, learner imbibes indirectly from slow channels of collateral experiences. sEMG learners can reinforce knowledge by a number of auxiliary learning methods such as interns at sEMG centers (where instrumentation is developed) alternatively learner can take up assignments like fellowships, apprenticeships, academic writing, review of articles and assisting teachers in preparing lectures in field of sEMG. In this way additional classroom load and assessment aspects are not built-up with the only scope of sEMG education and critical thinking. For implementing a robust ecosystem to promote sEMG learning, it is vital to understand importance of necessary competencies of trainer-trainee combination. Some suggested competencies are enlisted herein (but not limited to):

#### 5.3.1 Competencies of sEMG trainer

• Solid knowledge and teaching acumen of STEM topics.
• Sound understanding of relevant neuromuscular anatomy and physiology.
• Familiarity with common clinical situations of movement disorders to cite examples.
• Knowledge of neuromuscular diagnosis and assessment.
• Extra ordinary communication for translation of STEM topics in simpler form.
• Motivating and problem-solving personality to manage and steer learning goals.

#### 5.3.2 Competencies of a Trainee

• Clear concepts of basic physics and mathematics at senior secondary-education level.
• Understanding of basic neuromuscular anatomy and physiology required to apply sEMG.
• Motivation to practice sEMG with capacity to overcome sometimes frustrating difficulties.
• Ability to handle electrical instrumentation safely and diligently.
• Basic ability to operate computer software tools such as excel, python or matlab.

## 6 Discussion and conclusions

There is a widespread consensus about the need of reducing the gap between the available sEMG knowledge in the field of rehabilitation assessment and the clinical application of such technology in physiotherapy and movement sciences (2–4). There is also (a) extensive literature concerning the clinical applications of sEMG a sample is provided in (17–26), (b) the need to adopt evidence-based practice (EBP) approaches and (c) the need to increase consensus about sEMG-based planning and evaluation of interventions (2, 3, 15, 16). The need of translational efforts to increase the competence and technical expertise of clinical operators in these fields is also widely recognized. However, consensus on how to address these problems, how to disseminate knowledge and improve academic education, training and competence of professional clinical operators, is lacking. The benefits of sEMG are increasingly acknowledged, there is a critical need to increase its adoption. By enhancing the integration of sEMG into clinical practice, we can ensure a more structured approach to rehabilitation assessments, ultimately improving the effectiveness of interventions and patient outcomes.

## Appendix 1

### Glossary of terms

1. APTA, American Physical Therapy Association
2. BS, Bachelor of Science
3. MS, Master of Science
4. CME, Continuing Medical Education
5. HCPC, Health and Care Professions Council
6. ISEK, International Society for Electrophysiology and Kinesiology
7. JEK, Journal of Electromyography and Kinesiology
8. NCAHP, National Commission for Allied and Health Professions (Statutory commission in India)
9. NHS, National Health Service
10. PG, Postgraduate
11. PhD, Doctor of philosophy
12. PT, Physiotherapy/Physiotherapist
13. sEMG, Surface Electromyography
14. STEM, Science, Technology, Engineering, and Mathematics
15. UG, Undergraduate
16. WCPT, World Confederation for Physical Therapy
17. RMS, Root Mean Square

## References

[1] Merlo A Campanini I. Impact of instrumental analysis of stiff knee gait on treatment appropriateness and associated costs in stroke patients. Gait Posture. (2019) 72:195–201. 10.1016/j.gaitpost.2019.06.009 31228856

[2] McManus L Lowery M Merletti R Søgaard K Besomi M Clancy EA Consensus for experimental design in electromyography (CEDE) project: terminology matrix. J Electromyogr Kinesiol. (2021) 59:102565. 10.1016/j.jelekin.2021.102565 34102383

[3] Merletti R Temporiti F Gatti R Gupta S Sandrini G Serrao M. Translation of surface electromyography to clinical and motor rehabilitation applications: the need for new clinical figures. Transl Neurosci. (2023) 14(1):1–16. 10.1515/tnsci-2022-0279 PMC10024349 36941919

[4] Merletti R Disselhorst-Klug C Rymer WZ Campanini I. Surface electromyography: barriers limiting widespread use of sEMG in clinical assessment and neurorehabilitation. Frontiers Media SA. (2021) 12:1–4. 10.3389/fneur.2021.642257 PMC7906963 33643215

[5] The National Commission for Allied and Healthcare Professions Act. Official Website of Department of Health & Family Welfare, Government of Puducherry, India. Puducherry: Department of Health & Family Welfare, Government of Puducherry (2021). Available online at: https://health.py.gov.in/national-commission-allied-and-healthcare-professions-act-2021 (cited September 21, 2024).

[6] World Physiotherapy. Available online at: https://world.physio/ (cited September 21, 2024).

[7] American Physical Therapy Association | APTA. Available online at: https://www.apta.org/ (cited September 21, 2024).

[8] nhs.uk. NHS website for England. (2018). Available online at: https://www.nhs.uk/ (cited September 21, 2024).

[9] The Health and Care Professions Council (HCPC). Available online at: https://www.hcpc-uk.org/ (cited September 21, 2024).

[10] International Society of Electrophysiology and Kinesiology (ISEK). Available online at: https://isek.org/ (cited September 21, 2024).

[11] Clancy EA Morin EL Hajian G Merletti R. Tutorial. Surface electromyogram (sEMG) amplitude estimation: best practices. J Electromyogr Kinesiol. (2023) 72:102807. 10.1016/j.jelekin.2023.102807 37552918

[12] Del Vecchio A Holobar A Falla D Felici F Enoka RM Farina D. Tutorial: analysis of motor unit discharge characteristics from high-density surface EMG signals. J Electromyogr Kinesiol. (2020) 53:102426. 10.1016/j.jelekin.2020.102426 32438235

[13] Merletti R Cerone GL. Tutorial. Surface EMG detection, conditioning and pre-processing: best practices. J Electromyogr Kinesiol. (2020) 54:102440. 10.1016/j.jelekin.2020.102440 32763743

[14] Merletti R Muceli S. Tutorial. Surface EMG detection in space and time: best practices. J Electromyogr Kinesiol. (2019) 49:102363. 10.1016/j.jelekin.2019.102363 31665683

[15] Besomi M Hodges PW Van Dieën J Carson RG Clancy EA Disselhorst-Klug C Consensus for experimental design in electromyography (CEDE) project: electrode selection matrix. J Electromyogr Kinesiol. (2019) 48:128–44. 10.1016/j.jelekin.2019.07.008 31352156

[16] Besomi M Hodges PW Clancy EA Van Dieën J Hug F Lowery M Consensus for experimental design in electromyography (CEDE) project: amplitude normalization matrix. J Electromyogr Kinesiol. (2020) 53:102438. 10.1016/j.jelekin.2020.102438 32569878

[17] Klein CS Li S Hu X Li X. Editorial: electromyography (EMG) techniques for the assessment and rehabilitation of motor impairment following stroke. Front Neurol. (2018) 9:1–3. 10.3389/fneur.2018.01122 30619073 PMC6305436

[18] Drost G Stegeman DF van Engelen BGM Zwarts MJ. Clinical applications of high-density surface EMG: a systematic review. J Electromyogr Kinesiol Off J Int Soc Electrophysiol Kinesiol. (2006) 16(6):586–602. 10.1016/j.jelekin.2006.09.005 17085302

[19] Merlo A Bò MC Campanini I. Electrode size and placement for surface EMG bipolar detection from the brachioradialis muscle: a scoping review. Sensors. (2021) 21(21):7322. 10.3390/s21217322 34770627 PMC8587451

[20] Williams SE Koch KC Disselhorst-Klug C. Non-invasive assessment of motor unit activation in relation to motor neuron level and lesion location in stroke and spinal muscular atrophy. Clin Biomech Bristol Avon. (2020) 78:105053. 10.1016/j.clinbiomech.2020.105053 32563725

[21] Huppertz HJ Disselhorst-Klug C Silny J Rau G Heimann G. Diagnostic yield of noninvasive high spatial resolution electromyography in neuromuscular diseases. Muscle Nerve. (1997) 20(11):1360–70. 10.1002/(SICI)1097-4598(199711)20:11<1360::AID-MUS3>3.0.CO;2-8 9342152

[22] Munoz-Novoa M Kristoffersen MB Sunnerhagen KS Naber A Alt Murphy M Ortiz-Catalan M. Upper limb stroke rehabilitation using surface electromyography: a systematic review and meta-analysis. Front Hum Neurosci. (2022) 16:897870. 10.3389/fnhum.2022.897870 35669202 PMC9163806

[23] Xu Y Wang J Wang S Li J Hou Y Guo A. Neuromuscular conditions in post-stroke ankle-foot dysfunction reflected by surface electromyography. J NeuroEngineering Rehabil. (2024) 21(1):137. 10.1186/s12984-024-01435-5 PMC11304728 39107804

[24] Androwis GJ Pilkar R Ramanujam A Nolan KJ. Electromyography assessment during gait in a robotic exoskeleton for acute stroke. Front Neurol. (2018) 9:1–12. 10.3389/fneur.2018.00630 30131756 PMC6090052

[25] Boccia G Dardanello D Rosso V Pizzigalli L Rainoldi A. The application of sEMG in aging: a Mini review. Gerontology. (2015) 61(5):477–84. 10.1159/000368655 25502864

[26] Bashford J Mills K Shaw C. The evolving role of surface electromyography in amyotrophic lateral sclerosis: a systematic review. Clin Neurophysiol Off J Int Fed Clin Neurophysiol. (2020) 131(4):942–50. 10.1016/j.clinph.2019.12.007 PMC7083223 32044239

